# The function of APC/C^Cdh1 ^in cell cycle and beyond

**DOI:** 10.1186/1747-1028-4-2

**Published:** 2009-01-19

**Authors:** Min Li, Pumin Zhang

**Affiliations:** 1 Department of Molecular Physiology and Biophysics, Baylor College of Medicine, Houston, TX 77030, USA; 2 Department of Biochemistry and Molecular Biology, Baylor College of Medicine, Houston, TX 77030, USA

## Abstract

The anaphase promoting complex/cyclosome (APC/C) is a multi-subunit E3 ubiquitin ligase playing essential functions in mitosis. It is conserved from yeast to human and relies on two adaptor proteins, Cdc20 and Cdh1, to bring in substrates. Both APC^Cdc20 ^and APC^Cdh1 ^are implicated in the control of mitosis through mediating ubiquitination and degradation of important mitotic regulators such as cyclin B1, securin, and Plk1. In addition, APC^Cdh1 ^is thought to prevent premature S phase entry by limiting the accumulation of mitotic cyclins in G1 and to regulate processes unrelated to cell cycle. In this review, we will summarize our current understanding of APC^Cdh1 ^function in cell cycle and beyond.

## Introduction

Two ubiquitin E3-ligase complexes, SCF (Skp1/CUL1/F-box protein) and APC/C (anaphase promoting complex/cyclosome), control the timely transitions of cell cycle phases by promoting the degradation of many key cell cycle regulators. SCF complex mainly functions in G1, S and early M phases, whereas APC/C regulates mitosis including metaphase-anaphase transition and mitotic exit and maintains G1 phase [1,2]. APC/C is a large (1.5 MDa complex) composed of at lease 11 core subunits. It relies on two WD-40 repeat-containing adaptor proteins, Cdc20/fizzy(fzy)/p55CDC and Hct1/srw1/fizzy-related(fzr)/Cdh1, to engage with its substrates. Destruction box (RXXLXXXXN/D/E) and KEN box are motifs frequently found in APC's substrates, but other motifs are also possible for recognition by APC^Cdc20 ^or APC^Cdh1 ^[3]. The consensus sequence of destruction box can be found in many proteins. However, not all of these proteins are APC's substrates. Thus, there must be other sequence constrains we do not understand yet that define true APC substrates. Moreover, some substrates only have an RxxL motif and yet are recognized by APC, indicating the last amino acid in the consensus is not stringently conserved.

APC^Cdc20 ^initiates the metaphase-anaphase transition through mediating the ubiquitination and degradation of cyclin B1 and securin. To prevent premature separation of sister chromatids and mitotic exit, APC^Cdc20 ^is inhibited by Mad2 and BubR1 through the spindle assembly checkpoint mechanism [4-9]. Only when the sister chromatids are aligned at the metaphase plate and have established bivalent spindle attachment can the inhibition of APC/Cdc20 be released. In contrast to APC^Cdc20^, APC^Cdh1 ^is inactive in early mitosis [3] when it is inhibited by phosphorylation [10] and binding of Nup90/Rae1 complex [11,12]. APC^Cdh1 ^only becomes active from late mitosis to G1. The difference in the timing of activation between APC^Cdc20 ^and APC^Cdh1 ^suggests a functional division between the two E3 ubiquitin ligases in mitosis. Recent analyses of mice deficient in Cdc20 or Cdh1 strongly support that notion [13-15]. It appears that Cdc20 is required for metaphase to anaphase transition [15], whereas Cdh1 plays a nonessential role in mitotic exit but an essential role in G1/S regulation [13,14].

### Mitotic Function of Cdh1

A large number of mitotic regulators are degraded at the end of mitosis. These include Cdc20, Aurora B, Plk1, etc. and are most likely the substrates of APC^Cdh1 ^(Table 1) [16-43]. Although many of these mitotic regulators did accumulate in the absence of Cdh1, they were eventually degraded, probably because of the stabilization of Cdc20 that compensates for the loss of Cdh1 [13,14]. As a result, Cdh1-deficient cells could still proliferate. However, these cells did accumulate mitotic errors and display difficulties in completing cytokinesis, resulting in the formation of binucleated cells at a high frequency [13,14].

**Table 1:** Substrates of APC^Cdh1^.

Substrates	Function	Reference
Cdc20	anaphase onset	[16,17]
Securin	anaphase onset	[18,19]
Sgo1	anaphase onset	[20]
Rcs1	anaphase onset	[21]
XKid	spindle assembly	[22,23]
Tpx2	spindle assembly	[24]
Ase1	spindle assembly	[25,26]
Aurora A	mitotic exit	[27,28]
Aurora B	mitotic exit	[29]
Plk1	mitotic exit	[30]
Anillin	mitotic exit	[31]
CKAP2	mitotic exit	[32]
Cyclin B1	mitotic exit	[33]
Cyclin A	mitotic exit	[34]
Cdc6	DNA synthesis	[35-37]
Geminin	DNA synthesis	[38,39]
FoxM1	G1/G0 maintenance	[40,41]
SnoN	Tgf-β signaling	[42,43]
Ets2	Ras signaling	[77]
Id2	transcription	[86]

The lethality associated with the loss of Cdh1 is largely a result of failed development of placenta, an essential organ for embryonic life in mammals [13,14]. In the placenta, there is a special cell type, giant cells which are polyploid. The polyploidy is acquired through endoreplication. In the absence of Cdh1, the placental giant cells failed to form, suggesting that APC^Cdh1 ^is required for DNA endoreplication. To undergo endoreplication, a cell must not enter mitosis after a round of DNA synthesis. One critical factor for mitotic entry is cyclin B1, a substrate of APC^Cdh1 ^[33]. Therefore, it is possible that the failed formation of giant cells in Cdh1 mutant embryos was a result of the inability of the would-be giant cells to block the accumulation of cyclin B1. Indeed, cyclin B1 was readily detectable in the mutant placenta in the area where the giant cells should reside [14].

Like an endoreplicating cell, cells with their DNA damaged do not enter mitosis either. A recent study revealed that APC^Cdh1^-medicated degradation of Plk1 played an important role in preventing mitotic entry of DNA-damaged cells [44]. Under normal conditions, Cdh1 is kept inactive from late G1 to early mitosis. When cells suffered DNA damage, Cdc14B is translocated out of nucleoli into nuclearplasm where the phosphatase dephophorylates and activates Cdh1 [44]. It is unknown if a similar mechanism is employed by the placental giant cells to activate Cdh1 in G2 to prevent mitotic entry.

### Maintenance of G1 by APC^Cdh1^

In *Drosophila*, loss of *fzr *leads to an extra division cycle in the epidermis, a likely result of the accumulation of mitotic cyclins in G1 [45]. In HeLa cells, knocking down the expression of Cdh1 causes stabilization of Skp2 [46,47], a F-box containing protein responsible for bringing p27^Kip1 ^to the SCF complex for ubiquitination [48,49]. As a result, p27 is destabilized in the cells with impaired Cdh1 function and the G1 phase is shortened in these cells.

**Figure 1:**
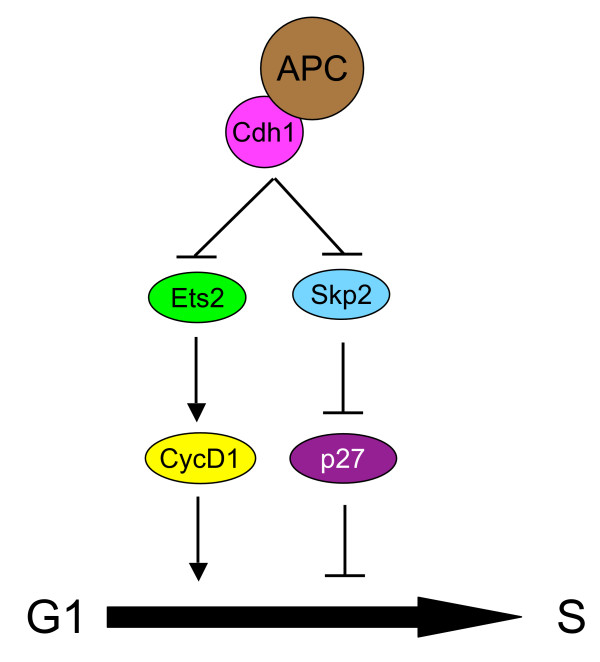
**Cdh1 regulate the timing of S-phase entry**.

Our recent work identified Ets2 as a new substrate of APC^Cdh1 ^[14]. Ets2 is a member of the Ets family of transcription factors which share a unique DNA binding domain, the ETS domain [50,51]. The first Ets protein was identified as transduced mutant form of Ets1, v-Ets, in a retrovirus, E26 (ETwenty six-specific, so the name Ets) avian leukosis virus (ALV), that induces erythroblastosis in avian species [52]. It is well known that Ets2 is activated by Ras-Raf-MAPK signaling and mediates some effects of this important signaling pathway [53-59]. The most prominent effect of Ras signaling is the stimulation of proliferation which relies in part on the induction of cyclin D1 expression by Est2 [60,61]. Increased expression of Ets2 has been associated with initiation and progression of various cancer types [62-65] and its expression was altered in cervical cancer cell lines due to chromosomal changes in 21q22.1–22.2 where human ETS2 resides [66]. Moreover, Ets2 was found overexpressed in esophageal squamous cell carcinoma [67]. These results suggest that Ets2 is an oncogene. By targeting both Skp2 and Ets2, APC^Cdh1 ^maintains G1 by increasing the levels of a Cdk inhibitor p27 through destabilizing Skp2 and by limiting the expression of cyclin D1 through promoting Ets2 degradation (Fig. 1). Since both Skp2 and Ets2 are potential oncogenes, APC^Cdh1 ^may possess tumor-suppression activity. Indeed, mice heterozygous for Cdh1 display increased rates of tumorigenesis at old ages [13].

It was shown previously by Nasmyth's group that inactivation of APC/C by deleting its subunit APC2 in adult hepatocytes induced these otherwise quiescent cells to re-enter cell cycle [68]. This is most likely a result of the loss of APC^Cdh1 ^activity and subsequent accumulation of Skp2 and Ets2.

To enter S-phase, APC^Cdh1 ^must be inactivated. Several different mechanisms are in place to contain APC^Cdh1 ^[69,70]. First, the ubiquitination of APC-specific ubiqutin-conjugating enzyme (E2) UbcH10 by APC^Cdh1 ^itself provides a negative feedback mechanism that would eventually destroy APC^Cdh1 ^activity [71,72]. Second, as Cdk activity accumulates, Cdh1 is phosphorylated. The phosphorylation promotes Cdh1 dissociation from APC [10,73]. Third, phosphorylated Cdh1 is targeted by SCF liagse [74], further limiting the activity of APC^Cdh1^. Finally, in late G1 phase, E2F activates the transcription of early mitotic inhibitor-1 (Emi1)/Rca1, which inhibits the activity of APC/C^Cdh1 ^as a pseudo-substrate [75,76].

### Cdh1 and Cellular Senescence

**Figure 2:**
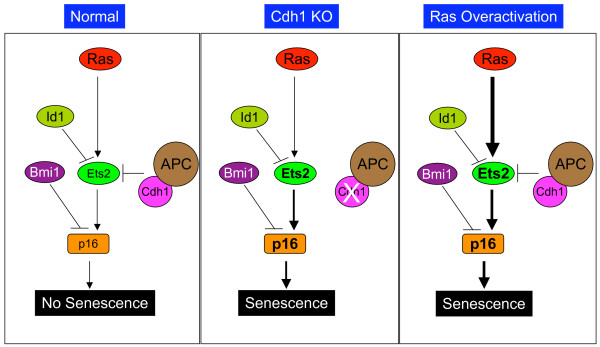
**Cdh1 and cellular senescence**. (A) Under normal conditions, the expression of p16 is tightly controlled by both positive and negative regulators. (B) Deletion of Cdh1 causes accumulation of Est2, leading to overexpression of p16 and senescence. (C) Oncogenic Ras may also induce senescence through Ets2.

Opposite to the expected high proliferation rates in Cdh1-deficient cells, Cdh1^-/- ^mouse embryonic fibroblasts (MEFs) proliferate poorly and entered senescence after only a few passages [77]. We found that the induction of p16^Ink4a ^in these cells was the cause for the reduced proliferation potential. The reason for the increased levels of p16 in Cdh1^-/- ^MEFs could simply be that p16 is a substrate of APC^Cdh1^. Alternatively, it could be that a transcriptional activator of p16 is a substrate. It turned out that the latter was the case [14]. We demonstrated that Cdh1-deficiency induced upregulation of p16 was a result of Ets2 stabilization. It was shown previously that Est2 could activate p16 expression [78]. The expression of p16 is regulated by a number of factors in response to various stimuli. Under normal conditions, Ets2 levels are not enough to induce p16 expression and senescence because of Id1 and Bim1 (Fig. 2A). Id1 interacts with Ets2 and blocks its transcriptional activation of p16 [78,79], whereas Bmi1 directly represses p16 promoter [80]. Our identification of the APC^Cdh1^-Ets2-p16 axis shows the delicacy of the balance that determines the level of p16 expression. Loss of Cdh1 leads to increases in the levels of Ets2 to the point that it overcomes Id1's inhibition and results in senescence (Fig. 2B). This would imply that Ets2 activity required for p16 activation and that for other targets are different, which remains to be determined.

Overactivation of Ras signaling could also cause senescence in primary cells [81-83]. It is likely that Ets2 mediates, at least in part, this senescence effect of Ras signaling (Fig. 2C). It remains to be determined however if Ras signaling interacts with APC^Cdh1^. The phosphorylation of Ets2 by Erk may interfere with the recognition of Ets2 by Cdh1, for example, leading to stabilization of Est2. Therefore, when Ras signaling is overactivated, APC^Cdh1 ^would only be able to provide limited balancing function against Ets2. By placing p16 under the transcriptional control of Ets2, evolution has setup a failsafe mechanism to prevent unwarranted proliferation.

### Cdh1 and Neural Function

Two places where appreciable levels of Cdh1 (and APC subunits) but not Cdc20 are expressed in adult mice are the brain and the liver [84]. In the liver, APC^Cdh1 ^is likely required for preventing hepatocytes from reentering the cell cycle spontaneously (see above). What is the function of APC^Cdh1 ^in postmitotic neurons? The depletion of Cdh1 expression in primary neuron of the cerebellar cortex promotes significant elongations of axons, indicating a role of APC^dh1 ^in limiting axonal growth [85], possibly through regulating the abundance of SnoN and Id2 [86,87]. APC^Cdh1 ^may also control pattering of axon growth in the mammalian brain [85].

We assessed neural functioning of mice that are heterozygous Cdh1 mutant. Electrophysiology studies of hippocampus revealed a deficit in late phase long-term potentiation (L-LTP), a process that underlies synaptic plasticity and is known to depend on both protein synthesis and degradation [88]. In behavior tests, we found that Cdh1 heterozygous mice performed poorly in contextual fear conditioning [14], a hippocampus-dependent process [89,90], but no difference was observed in cued fear condition which is less dependent on hippocampus [89,90]. Similar behavior findings were also reported by Malumbres' group [13]. It is unclear at the moment what are the substrates of APC^Cdh1 ^that play a role in memory formation in mammals. However, studies from lower organisms may provide a clue. In C. elegans, the abundance of GLR-1 glutamate receptors in the ventral nerve cord is regulated by anaphase promoting complex, albeit unlikely to be in a direct manner [91]. In Drosophila, Liprin-α, a multidomain scaffolding protein which is localized to synapses and regulates synaptic activities, seems to be a substrate of APC^Cdh1 ^[92]. It remains to be determined if mammalian homologues of Liprin-α and GLR-1 are regulated by APC^Cdh1 ^and if their misregulation can account for the learning and memory defects displayed by Cdh1 heterozygous mice.

## Conclusion

Tremendous progresses have been made towards understanding the regulation and function of APC/C in the last decade. Its role in cell cycle regulation has largely been elucidated. It appears that APC^Cdc20 ^is dedicated to mitosis while APC^Cdh1 ^has a much broader application not restricted to the cell cycle. The most intriguing question remains to be addressed is the role of APC/C in non-dividing cells. Accumulating evidence points to the involvement of APC^Cdh1 ^in many aspects of neural function including axon growth, morphology and plasticity of synapses, and learning and memory. Identification of the relevant substrates will provide significant insights into the functioning of the complex nervous system. Further, in addition to its potential role in maintaining the G0 state of hepatocytes, whether APC^Cdh1 ^participates in the physiological function of the liver remains to be elucidated

## Competing interests

The authors declare that they have no competing interests.

## Authors' contributions

ML and PZ wrote the manuscript together.
